# Current Positron Studies on the Modifications of the Molecular Packing in Green-Based Polymers Through Changes in the Synthesis Procedures or Environmental Conditions

**DOI:** 10.3390/polym16243611

**Published:** 2024-12-23

**Authors:** Giovanni Consolati, Carlos Macchi, Alberto Somoza

**Affiliations:** 1 Department of Aerospace Science and Technology, Politecnico di Milano, Via LaMasa, 34, 20156 Milano, Italy; 2 INFN Milan, Via Celoria, 16, 20133 Milan, Italy; 3 Positron Group “Prof. Alfredo Dupasquier”, Faculty of Exact Sciences, Tandil Institute of Materials Physics (IFIMAT), National University of the Center of the Buenos Aires Province (UNCPBA), Pinto 399, 7000 Tandil, Argentina; cmacchi@exa.unicen.edu.ar; 4 CIFICEN, UNCPBA-CICPBA-CONICET, Tandil B7000, Argentina

**Keywords:** carbohydrate polymers, free volume, green polymers, molecular organization, plant oil-based polymers, positron annihilation lifetime spectroscopy

## Abstract

The sensitivity of positron annihilation characteristics to changes in the molecular packing in network-forming polymers has been demonstrated since the early 1980s. Positron annihilation lifetime spectroscopy (PALS) is a unique technique that can provide direct information on the free volume in polymers through the experimental parameters of the free volume hole distribution, their mean value, and volume fraction. This knowledge is currently applied for PALS investigations on the main processes that govern the molecular organization in some green polymers when subjected to different synthesis procedures or environmental conditions (humidity, physical aging, temperature). In this article, which includes a wide repertoire of works published in the last two decades, results of PALS studies on eco-sustainable polymer systems based on starch, chitosan, or vegetable oils, are analyzed and discussed. Many examples are taken from the direct experience of the authors.

## 1. Introduction

Positron annihilation spectroscopy (PAS) is a well-established high-sensitivity technique for detecting open-volume sites in solids [1,2]. It has been applied to the study of the defect structure in solids for almost 50 years. Experimental variants of PAS, positron annihilation lifetime spectroscopy—PALS and Doppler broadening spectroscopy—DBS, have been used in many fields of materials science (for instance, cement [3], light alloys [4], polymers [5,6,7,8,9], and semiconductors [10,11]).

In conventional PAS experiments, positrons are implanted in a solid from a positron-emitting radioactive source with an initial kinetic energy well above the thermal energy. They then reach the thermal equilibrium in a few picoseconds. During this time, they penetrate a distance ranging from tens to thousands of microns, depending on the density of the solid. Therefore, in the mentioned experiments, positrons probe the bulk of the solid. The positron implantation time is short when compared to the time spent by the positron in the bulk before being annihilated, which in pure metals is typically above 100 ps [1]. Thus, the positrons used by PAS to probe a material are essentially thermal particles. In a perfect lattice, positrons survive in this free state until annihilation. Their mean lifetime, in this case, is determined by the electron density of the solid. However, positrons are repelled by electrostatic forces away from positive core ions; thus, any open volume available in the lattice (for instance, a vacancy, a void, or a lattice irregularity near to a dislocation) appears as a potential energy minimum for a positron. A quantum transition from the delocalized bulk state into a trapped state at a lower energy may occur. When a positron is trapped, annihilation takes place with the characteristics determined by the local electron density. The reduced electron density implies an increased positron lifetime, roughly between 30% and 70%; that is, trapped positrons survive for more time than the free ones. The number of positrons that annihilate follows an exponential decay law with time, with a characteristic half-life.

Trapping is the reason for the great sensitivity of positrons to defects. A positron in a solid ‘‘seeks’’ open-volume defects. For instance, a concentration of only one atomic ppm of vacancies in a pure metal already gives a distinct signal in a lifetime spectrum.

In the case of non-metallic solids, in addition to annihilation as a free positron, this last particle can form, with an electron of the medium, an unstable bound system, positronium (Ps). It is somehow a ‘minor brother’ of hydrogen, having a mass which is about one thousand times lower; the reduced mass, *m*_e_/2 where *m*_e_ is the electron mass, implies that the Ps energy levels are half those of hydrogen. In condensed matter, only Ps ground state is of interest, which is found in two sublevels: para and ortho-Ps, according to the spin orientations of the two particles (antiparallel and parallel, respectively). They show different features concerning lifetimes and annihilation: in a vacuum, para-Ps annihilates in two photons and has a lifetime of 125 ps. On the other hand, ortho-Ps (o-Ps) annihilates in three photons and its lifetime is 142 ns [12]. In a medium, Ps electron is repelled by the surrounding electrons due to exchange forces, and Ps tends to localize into opens spaces of the material hosting it. Thus, Ps is a ‘seeker’ of these open spaces, which in the case of polymers are identified as free volume holes. The para-Ps in a hole mainly annihilates with its own electron and the corresponding lifetime is scarcely influenced by the presence of other electrons. The case is different for o-Ps, which undergoes several interactions with the electrons belonging to the walls of the hole. Consequently, o-Ps can annihilate with an external electron in relative singlet spin state (‘pickoff’ process, with emission of two photons, instead of three), in addition to its own electron. The pickoff decay rate depends on the electron density, that is, on the size of the cavity, and in the case of sub-nanometric holes, reduces o-Ps lifetime by two orders of magnitude compared to vacuum. In free volume holes, o-Ps annihilation by pickoff is the dominant process.

Using a suitable model for the cavity, it is possible to quantitatively relate the o-Ps lifetime to the average size of the hole. In this frame, lifetime data can be transformed into average sizes of the free volume holes by using the Tao–Eldrup semiempirical equation [13,14]: the cavity hosting Ps is assumed to be a spherical hole with effective radius *R*. Such a Ps trap has a potential well with finite depth; however, for convenience of calculations, one usually assumes the depth is infinite, but the radius increased to R+ΔR, ΔR (0.166 nm) [15]. This empirical parameter accounts for the annihilation of o-Ps with the electrons belonging to the walls of the hole. Thus, the relationship between the lifetime *τ_o-Ps_* and the size of the hole is given by:

(1) $$\tau_{o-Ps}=0.5{\left[\frac{\Delta R}{R+\Delta R}+\frac{1}{2\pi }sin\left(2\pi \frac{R}{R+\Delta R}\right)\right]}^{-1}\;,$$

where *τ_o-Ps_* is given in ns. The values of the radii obtained from Equation (1) should be interpreted only as rough estimates, since real holes are irregularly shaped.

The average hole free volume *v*_h_ can then be calculated as

(2) $$v_{h}=\frac{4}{3}\pi R^{3}.$$

For holes larger than 1.5 nm, Equation (1) must be modified to account not only for the ground state but also for the excited levels of Ps inside the trap. This extended Tao–Eldrup model has been applied to spherical [16], rectangular [17], and cylindrical [18] holes. Within this framework, PALS has been used to study hole sizes up to tens of nanometers, a structure typical of mesoporous materials.

Concerning the relative number density of *v*_h_ in the material matrix, a simple approach considers it proportional to the intensity associated with the o-Ps lifetime *I_o-Ps_* [19,20]. Under this frame, the relative free volume *f_v_* can be written as:

(3) $$f_{v}=v_{h}I_{o-Ps},$$

where *o-Ps* is given in percentage [5]. This approach is valid when chemical processes involving Ps formation have no role. In fact, various factors influence Ps formation (and therefore *I_o-Ps_*) [21] and its contribution, due to the number density of holes, cannot be easily untangled. For example, *I_o-Ps_* obtained from PALS spectra measuring PVAc samples [22] over a wide range of temperatures show hysteresis, which has been explained in terms of radiation chemical processes in the terminal positron track [23]. In this case, it is difficult to correlate *I_o-Ps_* with the changes in the number density of holes with temperature. From a PALS study of γ-irradiated PE and PMMA6N (PMMA containing 6% methyl acrylate), Hirade et al. [24] reported that *I_o-Ps_* increases with time at low temperatures. The authors attributed this behavior to an increase in Ps formation in trapped electrons generated by irradiation. A quantitative analysis of this result [25] indicated that the probability of Ps formation is the main factor influencing *I_o-Ps_*, rather than just the number density of free volume holes, as all o-Ps are trapped before annihilation. The presence of positron acceptors (such as carbonyl groups) also influences the formation of positronium and, consequently, the value of *I_o-Ps_*. Therefore, this parameter is often the result of the contribution of several complex and interrelated processes [26], and Equation (3) should be considered with caution.

Furthermore, the distribution of hole sizes, typical of disordered structures, can be obtained from a distribution of o-Ps lifetimes using suitable statistics and careful analysis of time annihilation spectra. Therefore, positron annihilation as applied to polymers allows one to obtain valuable information about the free volume, a fundamental concept to explain many properties of these materials. Indeed, mechanical properties and free volume are negatively correlated [27], as an applied load does not distribute among the macromolecular segments but concentrates in the free volume, which increases the failure probability of the material. Also transport properties in membranes used in separation processes at the molecular scale depend on the free volume [28,29]. Physical aging in amorphous polymers below the glass transition temperature is another example involving free volume. After a prolonged period, the mass density increases with the corresponding reduction in the free volume [30].

Although hole free volumes can be examined by various probes, such as photochromic labels [31] and fluorescence molecules [32], the use of Ps to probe sub-nanometric holes offers several advantages: it is non-invasive, which allows the same sample to be used for other tests; it is smaller than the other probes and can better explore the lower tail of the hole size distribution; and finally, the instrumentation is rather simple.

The study of the free volume in polymers by PALS started in the early 60s and has dramatically grown since the availability of computer codes (around 1990) able to analyze the lifetime spectra of annihilations using a distribution of the mean lifetimes of the o-Ps, convertible into a distribution of hole sizes [33,34,35].

In recent decades, a huge interest in achieving sustainability from alternative eco-friendly resources produced a drastic change in fossil fuel-production, and its emphasis on petroleum has increased exponentially. Green materials are categorically eco-friendly and desirable due to their biodegradability, biocompatibility, and renewability. They are also sustainable and put less pressure on existing finite resources. Bio-based polymers are classified as any eco-friendly material that can be obtained from natural raw sources.

Previous insights into the importance of free volume can be extended to green polymers. To improve their mechanical and transport properties, among others, it is crucial to have information on the structure of polymers at the nanoscale, that is, their molecular organization. In this context, PALS provides valuable information to tailor polymer structures for specific applications.

Biodegradable polymers can be decomposed by microorganisms through enzymatic or chemical reactions, as long as the appropriate conditions are satisfied [36]. Depending on their origin, polymers can be classified as either natural or synthetic. Natural polymers originate mainly from biological or renewable resources (e.g., animals, plants, microorganisms), while synthetic polymers are created through chemical synthesis [37]. Important natural polymers are polysaccharides such as starch, cellulose, chitin, and its derivative chitosan, as well as protein-based polymers such as silk fibroin (SF), collagen, plant proteins, and vegetable-oils-based polymers.

Biodegradable synthetic polymers are characterized by ester, amide, or ether bonds; important examples include polylactic acid (PLA), polycaprolactone (PCL), poly(lactic-co-glycolic acid) (PLGA), polybutylene succinate (PBS), and polyvinyl alcohol (PVA) [38]. Most of the above-mentioned biodegradable polymers are also bio-resourced, making them important for a sustainable society. They are used in a great deal of practical fields. For instance, starch films find use as flexible and transparent substrates. Starch hydrogels can be processed into various shapes through 3D printing. Further carbonization allows one to obtain three-dimensional structured conductive materials. Many cellulose derivatives have been developed due to the ease of substituting its hydroxyl groups with functional groups. The high biodegradability of such compounds makes them ideal candidates to replace non-degradable polymers as dielectric materials.

Chitin is the second most abundant polymer in nature after cellulose, found in arthropods, mollusks, seaweed, and fungi. Chitosan, produced from the deacetylation of chitin, is a cationic natural polymer whose chemical properties are similar to cellulose. It is considered a widely applicable functional biomaterial [39], since its amino groups show high reactivity, which facilitates the performance of chemical modification. An important field in which chitosan finds use is water treatment. Indeed, amine groups can greatly help in the removal of heavy metal ions, which pose an environmental issue for water pollution [40]. These ions stem from a variety of widespread sources, such as mining waste [41], landfill leaches, domestic and industrial wastewater, especially from the electroplating, electronic, and metal finishing industries [42]. Their non-degradability, combined with accumulation within the body of humans and animals, is a major issue as it is extremely hazardous, even at low concentrations.

Plant oils are a renewable resource that can be used to formulate new biobased functional polymeric materials with diverse structural and functional properties. Their abundance and affordability make them attractive as raw materials for the plastics industry [43,44,45].

Plant oils and their derived fatty acids are among the most important renewable feedstocks in the chemical industry due to their availability, inherent biodegradability, low price, and low ecotoxicity. Major sources of vegetable oils include palm trees, soybeans, rapeseeds, cotton, sunflower, palm kernel, olives, and coconuts. Triglycerides, composed of glycerol esterified with three fatty acids, are the primary constituents of plant oils. Fatty acids make up about 95% of the total weight of triglycerides and vary in composition among different plant oils. The double bonds and ester groups in triglycerides offer numerous opportunities for structural modification.

Thermoplastic and thermosetting materials can be derived from triglycerides and their derivatives. However, most plant oil-based thermoplastics and thermosets exhibit long-chain polymer characteristics, such as high elongation at fracture and relatively low stiffness [46]. To address these limitations, this kind of material is often blended with synthetic comonomers to create composites. Vegetable oil derived polymers/composites are used in diverse applications such as paints and coatings, adhesives, foams, and shape memory materials [47].

Recently, taking advantage of the well-recognized capability of the PALS nuclear technique to provide direct information on hole free volumes, a research focus on green polymers using PALS has emerged. Particularly, this work addressed the study of some natural ecopolymers; (i) starch-based; (ii) chitosan-based; and (iii) vegetable-oils based polymers. These natural resources were chosen due to their importance in the field of biodegradable polymers, as well as to the need to limit the field of the present review because it would become too broad to be adequately treated in this article.

## 2. Experimental Setup

This paragraph is intended for readers unfamiliar with PALS; further information can be found in the literature, e.g., [48,49]. PALS consists of injecting positrons into a sample and measuring their lifetime spectrum. In most applications, the positron source is the *ß^+^* radioactive isotope ^22^NaCl with activity between 0.04 and 1 MBq. It is deposited onto two thin metallic or plastic foils (~a few μm) and sealed to prevent environmental contamination, allowing for multiple experiments. The positron source is then located between two identical specimens, forming a ‘sandwich’ geometry. More than 99% of the injected positrons have to be annihilated in the sample. For polymers, a thickness of 1 to 2 mm is sufficient for this purpose.

The advantage of the ^22^Na positron source is that its most frequent decay channel is the emission of a photon of 1.274 MeV in coincidence with the positron. This property is very useful, as the 1.274 MeV photon signals the birth of the positron. The annihilation signal consists of one of the two 0.511 MeV photons, resulting from the conversion of the annihilated particles’ mass into electromagnetic energy.

**Figure 1:**
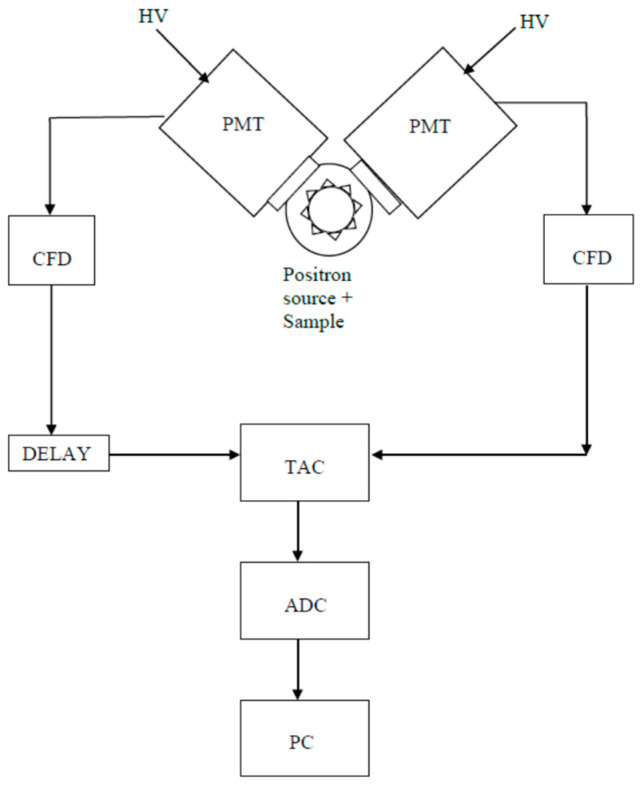
Simplified block diagram of a PALS spectrometer.

A simplified block diagram of a two-channel PALS spectrometer is shown in Figure 1. Each channel consists of a scintillator (either organic or fast inorganic, like BaF_2_) coupled with a photomultiplier tube (PMT). When a γ-ray with the correct energy (either the 1.274 MeV for the start signal or the 0.511 MeV for the stop signal) is detected, a fast signal is generated by the constant fraction discriminator (CFD). A time-to-amplitude converter (TAC) triggered by the ‘start’ CFD generates a voltage linearly increasing with time, and it is terminated by the ‘stop’ signal. The TAC output is a pulse whose height is proportional to the time delay between start and stop signals. The signal is digitized by an analog-to-digital converter (ADC) and transferred to a personal computer (PC).

Since the early 2000s, PMT signals have been digitized using ultra-fast modules, replacing some components (like CFDs and TACs) in the apparatus [50,51]. Consequently, time resolution has improved compared to standard configurations, without sacrificing counting rate [52].

Thin polymer films and polymer surfaces can also be investigated using positrons. For this purpose, a monoenergetic slow positron beam must be used. The energy of the positron beam can be tuned from a few eV to several keV [53,54], allowing control over the depth of positron implantation in the materials. We primarily used the information on positron annihilation in polymer films and surfaces provided by Doppler broadening spectroscopy (DBS). However, positron annihilation lifetime spectroscopy (PALS) has also been employed [55,56].

A positron annihilation lifetime experimental spectrum is the convolution of an intrinsic spectrum (a histogram with millions of counts) with the resolution function (the apparatus’s response to two simultaneous events), typically ranging from 150 to 350 ps. A spectrum is analyzed using suitable computer code with various programs widely available for free [33,34,35,57].

A spectrum is the sum of discrete and/or continuous lifetime components, each corresponding to a specific positron state. In the discrete case, a component is represented by an exponential decay function $\frac{I}{\tau }exp\left(-t/\tau \right)$, characterized by a lifetime *τ_i_* and intensity *I_i_*. Thus, a PALS spectrum *S*(*t*) can be represented as follows:

(4) $$S(t)=R(t)⊗[\sum_{i=1}^{N}\frac{I_{i}}{\tau_{i}}\exp(-t/\tau_{i})+B]$$

*R*(*t*) is the resolution function, and *B* is the background, which must be subtracted during the fitting process. In polymers, analyses using three lifetime components are quite common.

In a polymer, a free positron that does not form positronium (Ps) typically has a lifetime of ~0.4 ns. This relatively short lifetime is assigned to the high probability of positron annihilation with electrons in the polymer matrix. Para-positronium has a very short lifetime, usually around 0.15 ns, whereas o-Ps shows the longest lifetimes, generally between 1 and 10 ns. In some polymers, two long-lived components are sometimes found in PALS spectra.

In a PALS spectrum, a continuous long-lived lifetime component can be modeled as a continuous sum of discrete components, each characterized by three parameters: intensity and the first and second moments of the lifetime distribution (centroid and standard deviation from the mean lifetime, respectively). In a polymer, an o-Ps lifetime distribution is expected to reflect the hole-free volume distribution. To accurately resolve continuous components in a PALS spectrum, significantly higher statistics are needed compared to discrete component analysis.

## 3. Results

### 3.1. Starch-Derived Systems

Several research groups have investigated the effects of various parameters linked to synthesis procedures (basically, blend composition and water content), and/or environmental conditions (aging time, temperature, ambient moisture, and pressure) on the molecular packing in carbohydrate polymers matrices [58,59,60,61,62,63,64,65,66,67,68,69,70].

#### 3.1.1. Maltodextrin-Based Matrices

Diverse techniques have been developed to stabilize and protect sensitive bioactive compounds for food and pharmaceutical applications [71]. Among these techniques, encapsulating the bioactive compound in amorphous carbohydrates in the glassy state is a particularly promising strategy. Freeze-drying or spray-drying concentrated carbohydrate solutions is a common technique for creating these encapsulating materials. The molecular mobility within the carbohydrate matrix significantly influences barrier properties, protecting the bioactive compound from degradation by external factors like oxygen and water. Adding specific low-molecular-weight compounds to higher-molecular-weight carbohydrates can enhance storage stability and lower the glass transition temperature. Effective combinations of plasticizers and carbohydrates, such as sucrose–maltodextrin and low-molecular-weight starch hydrolysates (glucose, maltose, and maltotriose) with maltodextrin, have been identified [72].

**Figure 2:**
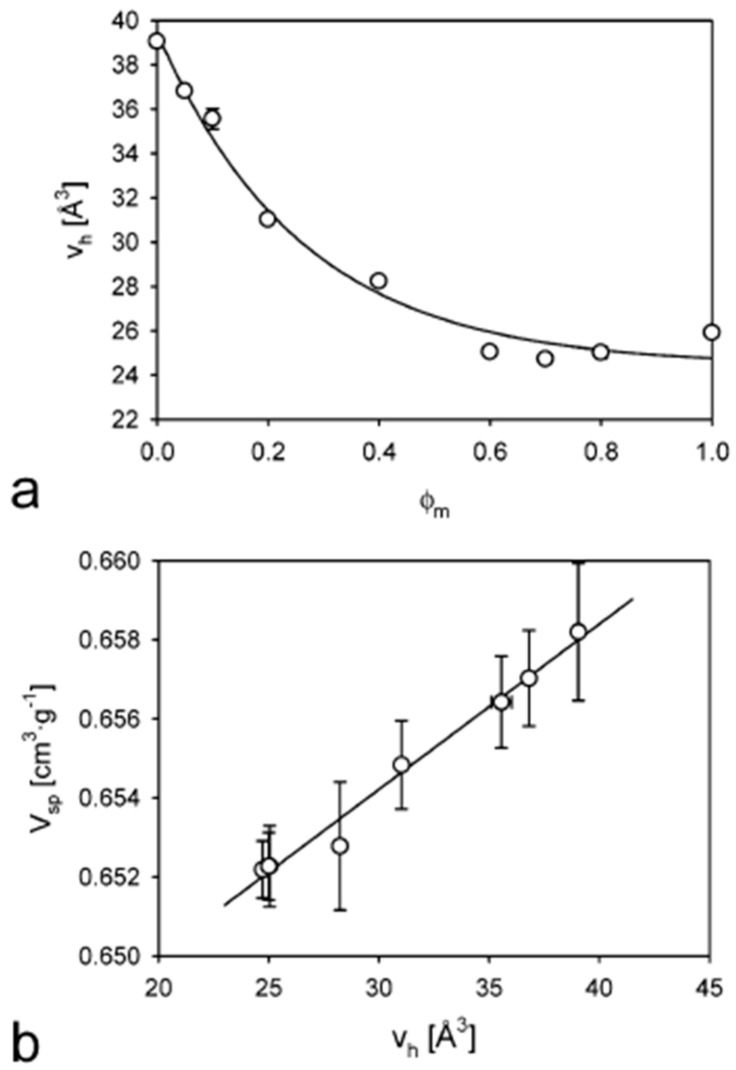
(**a**) Free volume hole size as a function of φ_m_, on anhydrous carbohydrate basis. All matrices are equilibrated at a_w_ = 0.33 and T = 25 °C. The solid line is an exponential fit of the data. (**b**) Relation between the hole size and the specific volume of the glassy maltopolymer–maltose matrices at a_w_ = 0.33 and at room temperature (for more details see [60]) (reprinted with permission from {Townrow, S., Kilburn, D., Alam, A., Ubbink, J., Molecular Packing in Amorphous Carbohydrate Matrixes, *J. Phys. Chem. B* **2007**, *111*, 12643–12648}. Copyright {2007} American Chemical Society).

The Bristol Positron Group and Nestlé Research Center have extensively studied the interactions between water and amorphous polysaccharide matrices [58,59,60,61,62]. In particular, the authors investigated the influence of various parameters, including water content, carbohydrate molecular weight distribution, and temperature, on the nanoscale molecular packing structure of carbohydrate matrices such as maltodextrin, maltopolymer–maltose blends, and maltodextrin–glycerol blends (0–0.20 glycerol weight fraction). These investigations were primarily conducted using Positron Annihilation Lifetime Spectroscopy (PALS) and Pressure–Volume–Temperature (PVT) analysis. Depending on the specific research objective, additional conventional techniques such as Differential Scanning Calorimetry (DSC), density measurements, water activity and content determinations, and dilatometry were employed. The combination of these experimental techniques provided a comprehensive understanding of the molecular-level mechanisms responsible for the observed changes in specific volume and hole free volume within the carbohydrate matrices. For instance, the authors observed a non-linear relationship between the weight fraction of maltose φ_m_ and the average size of free volume holes in glassy maltopolymer–maltose blends maintained at a constant water activity a_w_ = 0.33 and room temperature (see Figure 2a). Furthermore, a linear relationship was obtained between the average hole size and the specific volume of the matrices, as illustrated in Figure 2b. This finding establishes a direct correlation between a molecular-scale parameter (hole size) and a macroscopic property (specific volume).

**Figure 3:**
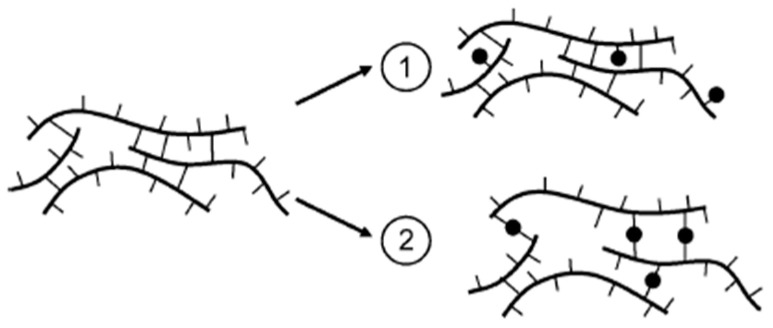
Schematic depiction of the mechanism of sorption of water by amorphous carbohydrates. (for more details see [59]) (reprinted with permission from {Kilburn, D., Claude, J., Schweizer, T., Alam, A., Ubbink, J., Carbohydrate Polymers in Amorphous States: An Integrated Thermodynamic and Nanostructural Investigation, *Biomacromolecules*, **2005**, *6*, 864–879}. Copyright {2005} American Chemical Society).

On the other hand, the results reported by Kilburn et al. in [59] revealed that water sorption in amorphous carbohydrate matrices is a complex process involving the formation and disruption of hydrogen bonds, as well as changes in the free volume of the matrix. At low water contents, water molecules act as hole fillers, potentially contributing to antiplasticization effects. At higher water contents, corresponding to water activities above 0.11 at 25 °C, water molecules can disrupt the hydrogen bonding network between carbohydrate molecules, leading to an increase in the average hole free volume and local expansion of the molecular packing structure. In Figure 3, the authors show a smart schematic representation of the two principal hydrogen bonding mechanisms between water and carbohydrate chains. In Case 1, water sorption occurs without substantial matrix swelling, resulting in densification and a reduction in the hole free volume. In Case 2, water adsorption occurs with substantial matrix swelling, even in the glassy state, leading to an increase in average hole size. Amorphous carbohydrate polymers exhibit behavior that lies between the two limiting Cases 1 and 2.

Additionally, S. Townrow et al. [61] investigated the influence of temperature, water content, pressure, and blend composition on the specific volume and nanostructure of the hole free volume in amorphous blends of maltose with a narrow molecular weight distribution maltopolymer. Results from PALS and PVT measurements in both the glassy and rubbery phases, as well as in solution, revealed a linear correlation between hole free volume and specific volume, with a more pronounced increase observed in the rubbery state compared to the glassy state. Remarkably, no discontinuity was observed in either specific volume or hole volume at the glass transition temperature. As the maltose content increased, a decrease in both specific volume and hole volume was observed in the glassy state, suggesting a more efficient molecular packing arrangement. A similar linear relationship between hole free volume and specific volume was observed with changes in carbohydrate composition.

Water content had a more complex effect. While increasing water content generally decreased specific volume in both states, this behavior was attributed to the dispersion of water molecules within the glassy carbohydrate matrix, likely influencing hydrogen bonding interactions between carbohydrate molecules.

In contrast, it was reported that glycerol acted as a packing enhancer in maltodextrin-glycerol blends, reducing molecular hole size. This effect was similar to that of maltose, but with a more limited concentration range. Water exhibited complex behavior in these blends, acting as an anti-plasticizer at low concentrations, which reduced molecular mobility, and as a plasticizer at higher concentrations, increasing free volume. This transition was attributed to the interplay between the ability of water molecules to form hydrogen bonds with the carbohydrate matrix and their impact on molecular mobility.

#### 3.1.2. Starch-Sucrose Blends

Within the collaborative framework between the Bristol Positron Group and researchers from Swiss private food companies, focused on developing sustainable food concepts, fundamental research on various hydrophobically modified starch (HMS) and sucrose (S) blends was carried out. Specifically, HMS–S blends were synthesized with sucrose content ranging from 0 to 40 wt.%, and then embedded in a shell of corn starch particles. To understand how different structural aspects of these blends impact their potential use in actual encapsulation systems, Hughes et al. [63] reported results on the dependence of the local free volume of HMS–S blends on both blend composition and water content (water activity a_w_ between 0.11 and 0.75). For their investigation, the authors used wide-angle X-ray scattering (WAXS) combined with PALS. PALS spectra were analyzed by assuming a distribution in the o-Ps component; in this case, describing the o-Ps distribution are *τ_o-Ps_* and *σ_o-P_*, respectively. Here, *σ_o-Ps_* represents the dispersion in the lifetime distribution and, consequently, the dispersion in the hole size distribution.

**Figure 4:**
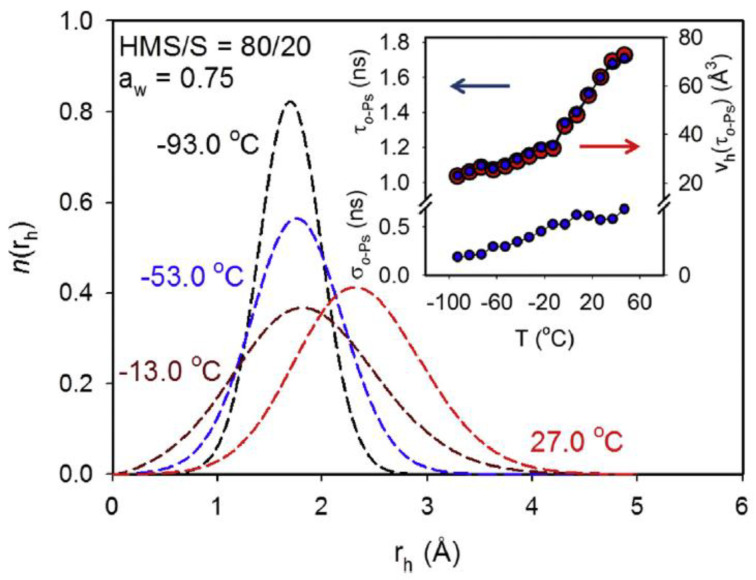
Probability distribution of hole radii for the HMS/S = 80/20 sample, equilibrated at a_w_ = 0.75; measurements were carried out at different temperatures. The inset presents the variation in the average values of *τ_o-Ps_, σ_o-P_*, and the calculated average *v_h_* as a function of temperature (reproduced from ref. [63] with permission. Copyright {2016} from Elsevier).

In Figure 4, the probability distributions of hole radii *n*(*r_h_*) for the HMS–S = 80–20 sample equilibrated at a_w_ = 0.75 at different temperatures are shown. Variations in the average o-Ps lifetime, dispersion, and the calculated average hole volume as a function of temperature, respectively, are shown in the inset of the figure.

WAXS results indicated that HMS–S blends were almost amorphous, with a low degree of crystallinity, while PALS studies implied that the hole free volume decreases with increasing sucrose content for all blends. This behavior was assigned to two factors: (i) the S molecules in the HMS-rich phase reduce the average size of the hole free volume, via a mechanism similar to that observed by [60,61,62] in other blends consisting of a carbohydrate matrix with a low-molecular-weight sugar. The role of sugar in these blends is reflected in a decrease in the glass transition temperature, thereby extending the high thermal expansivity of the rubbery state of the blend to lower temperatures, leading to a denser molecular packing; and (ii) the average hole size in the S-rich phase is smaller than that in the HMS-rich. An increase in the S content in the blend increases the fraction of the S-rich phase, thus reducing the value of *v_h_*. Additionally, it was reported that the temperature dependence of *τ_o-Ps_* reflects the phase separation behavior, with distinct slopes according to the *T*_g_ of the respective phases. In the mentioned articles, the authors also studied the effect of water content on the local free volume, finding that this change is significant. Furthermore, it was found that at low-water activities, the presence of water leads to a decrease in the average size of *v_h_*. This behavior agrees with the anti-plasticization regime, in which water molecules occupy positions at the edges of the holes, reducing their sizes. However, as water content exceeds a certain threshold, the HMS–S blend was observed to switch to a plasticizing regime, where *v_h_* starts to increase again. This behavior was attributed to enhanced local molecular motions, as water replaces interchain hydrogen bonds with water–chain hydrogen bonds, allowing for greater mobility and larger free volumes. Given the high heterogeneity of HMS–S blends consisting of phase-separated systems, it was also highlighted in the aforementioned study that water content influences the dispersion of the hole volume distribution.

As part of the same broad research collaboration between the Bristol Positron Group and researchers in chemistry and food science, Martini et al. [68] investigated the same HMS–S blends studied by Hughes et al. [63]. But in this case, the goal of the work was focused on understanding the molecular mechanisms underlying amorphous–amorphous phase separation. To this aim, experimental studies were carried out using PALS and solid-state nuclear magnetic resonance (NMR) simultaneously. The results indicated that sucrose acts as an anti-plasticizer for HMS. Furthermore, selective solid-state ^13^C NMR experiments revealed that a significant portion of the sucrose molecules are in direct contact with the HMS, even at high concentrations of S. From these results, the authors concluded that the phase separation between HMS and S was not completed. Further analysis showed that sucrose migrates away from the HMS-rich domains at temperatures above the lower *T*_g_ but below the upper one. Additionally, selective solid-state ^13^C NMR experiments revealed that a significant portion of sucrose molecules were in direct contact with HMS, even at high concentrations of S. From these results, the authors concluded that the phase separation between HMS and S was incomplete. Moreover, ^1^H spin diffusion experiments indicated that phase separation occurs on a nanometric scale, aligning with recent theoretical findings [73]. In summary, these results revealed that there was a complex phase behavior in these blends. Water and low molecular weight compounds could easily migrate between different phases within the material, affecting the physical stability and barrier properties of the blends [62].

#### 3.1.3. Thermoplastic Starch-Based Films

When combined with water and plasticizers (such as glycerol and sorbitol), starch granules can be processed to produce a biodegradable polymer known as thermoplastic starch (TPS). Recently, Estévez Areco et al. [70] reported results from a study on the influence of citric acid (CA) concentration and water content on the free volume of thermoplastic cassava starch films. To gain detailed insights into how CA concentration affects TPS films (~0.5 mm thick), continuous in situ PALS measurements were performed on samples with CA concentrations ranging from 0 to 5% (in *w*/*w* to starch).

First, the structural characteristics of the TPS-CA films were determined under controlled equilibrium humidity conditions (RH = 58%). The samples were subsequently exposed to a drier atmosphere (RH = 20%) to monitor changes in the hole size distribution during water desorption.

**Figure 5:**
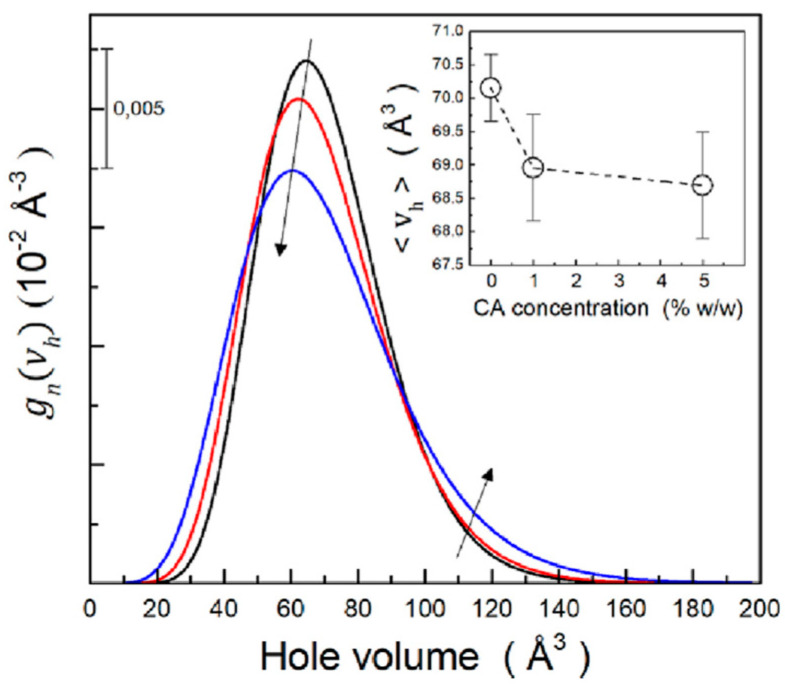
Hole volume distributions for TPS (black line), TPS-CA1 (red line), and TPS-CA5 (blue-line) films stabilized at RH = 58%. The inset shows the mean hole volume as a function of CA concentration (reproduced from ref. [70] with permission. Copyright {2023} from Elsevier). Arrows indicate the behavior of the distribution at increasing CA concentration.

The PALS results were combined with data obtained from water susceptibility and mechanical property measurements. The authors observed that increasing the CA concentration in TPS films led to a broader distribution of hole free volumes, coupled with a reduction in the mean free volume size. This effect can be observed in Figure 5.

The decrease in mean free volume sizes was associated with the formation of ester bonds between citric acid and starch chains, which limited chain mobility. Conversely, the increase in the width of the hole volume distribution with higher CA content suggests a more open network structure, possibly due to the higher water content of this material.

**Figure 6:**
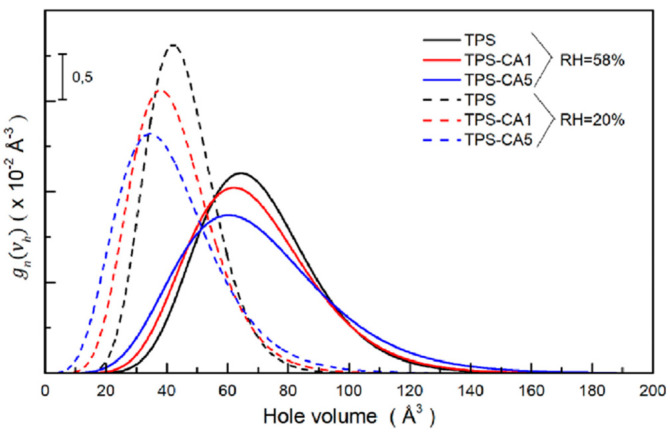
Hole volume distributions for the TPS, TPS-CA1, and TPS-CA5 films stabilized at RH = 58% (continuous lines) and RH = 20% (dashed lines) (reproduced from ref. [70] with permission. Copyright {2023} from Elsevier).

Regarding the water desorption process, the authors observed that it significantly reduced the mean hole volume and narrowed the hole volume distributions, indicating a decrease in the plasticizing effect of water content due to structural changes, as shown in Figure 6. This process stabilized after 100–120 h, leading to a more compact structure, increased stiffness, and reduced flexibility. Additionally, Young’s modulus for all films increased substantially after partial water desorption. This was attributed to the loss of water acting as a plasticizer, leading to a reduction in the mean hole volume. Again, PALS gives a microscopic interpretation of this effect. Indeed, the removal of water, which acts as a plasticizer, leads to a reduction in the mean hole volume, which is negatively correlated to the tensile modulus [74,75]. These findings emphasize the necessity of controlling relative humidity during testing, as the nanostructure of TPS-based films is highly sensitive to external conditions.

### 3.2. Chitosan

Despite its abundance, versatility, and biodegradability, chitosan has limitations like high moisture absorption, low processing temperature, heat instability, and flammability. To overcome these challenges and broaden its applications, researchers have explored various strategies, including crosslinking, graft copolymerization, complexation, chemical modifications, and blending.

Based on the nature of the co-polymer, these derivatives can be categorized into natural chitosan-based polymers and synthetic chitosan-based polymers. The former group includes chitosan combined with naturally occurring polymers such as proteins (collagen, silk), polysaccharides (alginate, cellulose), or nucleic acids, while the latter consists of chitosan combined with synthetic polymers.

Although this area has attracted considerable attention in recent years, to our knowledge, only a few studies have focused on the study of chitosan-based polymers using PALS as the main experimental technique [76,77,78,79,80,81,82,83,84,85,86,87,88,89,90,91].

#### 3.2.1. Pure Chitosan

Chitosan’s (CS) physical, chemical, and biological properties are closely linked to its molecular structure, including its degree of deacetylation, molecular weight, crystallinity, and functional group distribution. Many of its limitations, such as low water solubility and high viscosity, are also structure related.

By modifying the structure of chitosan, it is possible to improve its processability and expand its range of applications. For instance, Lucanera et al. [88] studied the effects of deacetylation on the physicochemical, thermal, and nanostructural properties of pure β-CS derived from squid pens during the deacetylation process. In their studies, a variety of analytical techniques were utilized, including potentiometric titration, capillary viscosimetry, Fourier Transform Infrared Spectroscopy, Differential Scanning Calorimetry, and Positron Annihilation Lifetime Spectroscopy. 

**Figure 7:**
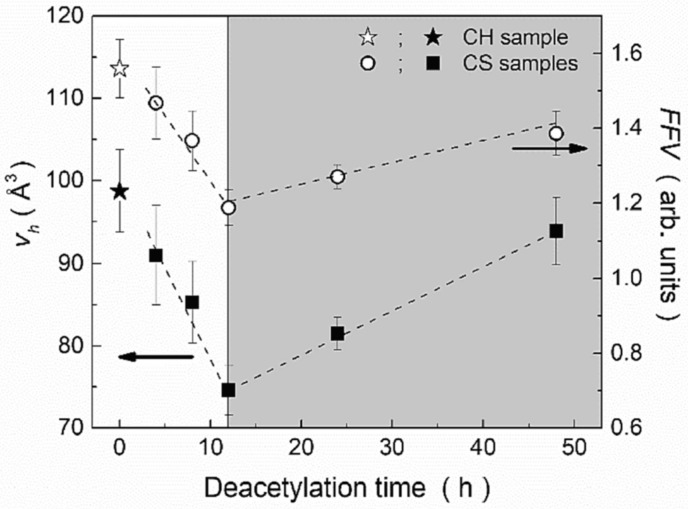
Free volume holes and fractional free volume as a function of deacetylation time for pure chitin and deacetylated chitosan. The dotted lines are provided solely as a visual aid (reproduced from ref. [88] with permission. Copyright {2022} from Elsevier).

Key results from this study indicated that the deacetylation process significantly increased the degree of deacetylation (DD%) within the initial 12 h, reaching approximately 80%, and then increased more gradually, achieving values between 90% and 98% after 48 h. Concurrently, the molecular weight decreased markedly to 184 kDa after 48 h. FT-IR analysis confirmed reduced acetamide groups and increased amine groups, aligning with deacetylation. DSC thermograms revealed shifts in thermal degradation peaks corresponding to amine and acetamide groups, reflecting structural changes. PALS measurements revealed an initial decrease in both the average hole free volume and fractional free volume during the first 12 h of deacetylation. However, these parameters increased with prolonged treatment times, as presented in Figure 7. Finally, from the results obtained, it could be concluded that the nanostructure of chitosan can be tailored by varying the deacetylation time, allowing for the preparation of chitosan matrices with specific nanostructural characteristics, which are crucial for applications such as drug delivery and pollutant adsorption systems. The non-monotonous behavior of hole sizes with increasing deacetylation times suggests that chitosan matrices with different DD% and molecular weights can be achieved, fulfilling specific application requirements.

Additionally, the capacity of pure chitosan as a heavy metal adsorbent was studied, along with the impact of metal adsorption on chitosan’s molecular packing structure. Anbinder et al. [85] studied structural changes in commercial chitosan films after Cu(II) and Cr(VI) ion adsorption during the soaking of the chitosan films in different concentrations of these metal ions, employing various experimental techniques, including FT-IR, UV-vis spectroscopy (UV-vis), DSC, TGA, and PALS.

**Figure 8:**
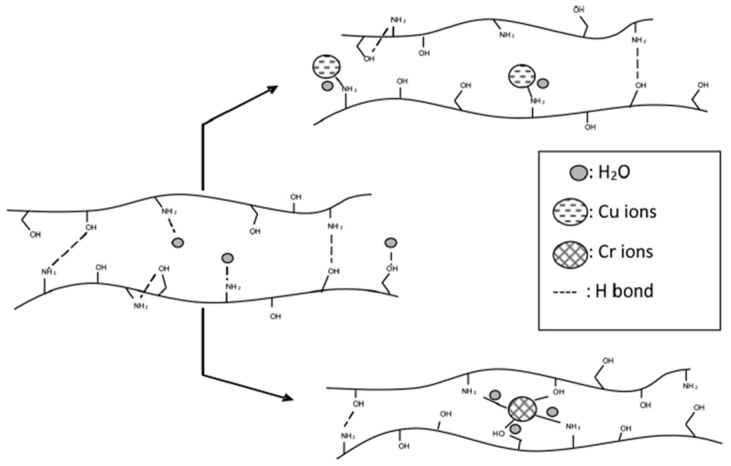
Schematic illustration of the chitosan structure and metal ion interactions (reproduced from ref. [85] with permission. Copyright {2019} from Elsevier).

Key findings include enhanced Cu(II) sorption compared to Cr(VI), as evidenced by colorimetric changes. UV-vis spectroscopy confirmed metal complex formation, supported by FT-IR analysis. Thermal analysis revealed that Cu(II) adsorption reduced thermal stability, while Cr(VI) adsorption increased it. PALS showed decreased hole free volume, especially for Cr(VI) adsorption. Smaller hole sizes suggest increased inter- and intra-chain interactions, reducing chain mobility. DSC and FT-IR support multiple interactions between Cr ions and amine/hydroxyl groups, leading to increased rigidity, in agreement with PALS results. In contrast, Cu ion adsorption leads to larger hole sizes and a more flexible matrix, likely due to pendant binding involving a single amine group, as sketched in Figure 8.

These findings provide valuable insights into metal–chitosan interactions, facilitating the design of more effective biopolymeric systems for environmental remediation.

#### 3.2.2. Natural Chitosan-Based Polymers

Abdel Hady et al. [91] studied the effects of blending α-CS and β-CS with starch and glycerol on the physical properties of chitosan films for biodegradable packaging. Techniques like PALS, XRD, and SEM were employed to analyze the films. The films were prepared by blending chitosan with starch at concentrations ranging from 20% to 80%, with 1% glycerol added to improve flexibility. The films were cast onto Teflon plates and dried under controlled conditions.

Pure β-CS exhibited smaller, more numerous hole free volumes than α-CS, likely due to differences in chain bonding. The addition of starch (20–50%) reduced hole size and increased their fraction, suggesting increased chain packing density.

In both chitosan types, glycerol further increased the free volume, suggesting increased flexibility. XRD showed decreased crystallinity at higher concentrations of starch, while SEM revealed varying morphologies between chitosan types. The study concluded that starch significantly influences the free-volume characteristics of chitosan films, with effects depending on the chitosan type.

Lin et al. [79] prepared starch-based composite materials (STM) using cassava starch and modified cellulose, then coated them with chitosan to improve hydrophobicity and thermal resistance. This study aimed to address the limitations of starch-based materials in packaging and biomedical applications. Techniques like FTIR, SEM, Polarized Optical Microscopy (POM), AFM, PALS, and mechanical testing were used to characterize the composite materials. The chitosan coating increased the STM’s tensile strength from 3.48 to 6.77 MPa. The coating reduced water absorption and dissolution, improving water barrier properties. PALS showed that the chitosan coating stabilized the hole free volumes of STM during vapor heat treatment, indicating enhanced water resistance and stability.

In conclusion, the chitosan coating successfully enhanced the mechanical properties, water barrier properties, and thermal resistance of starch-based composite materials.

#### 3.2.3. Synthetic Chitosan-Based Polymers

**Figure 9:**
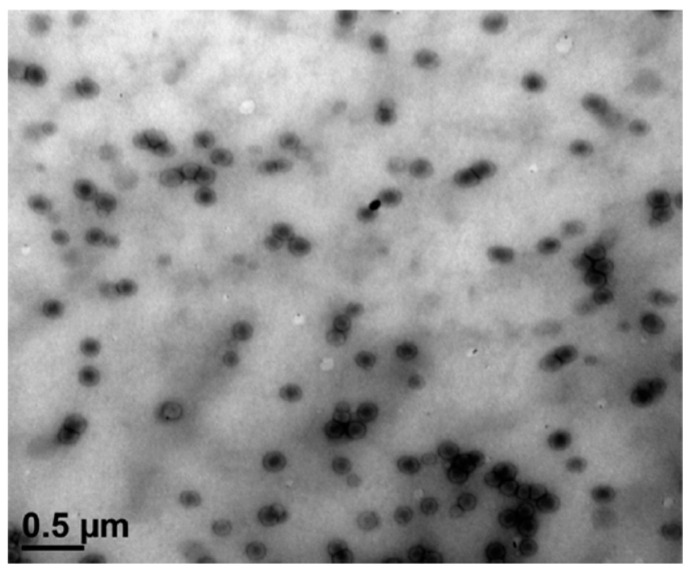
TEM micrograph of a dispersion corresponding to the QSA50 grafted sample. A more detailed description of the samples is available in ref. [81] (reproduced from ref. [81] with permission. Copyright {2016} from Elsevier).

**Figure 10:**
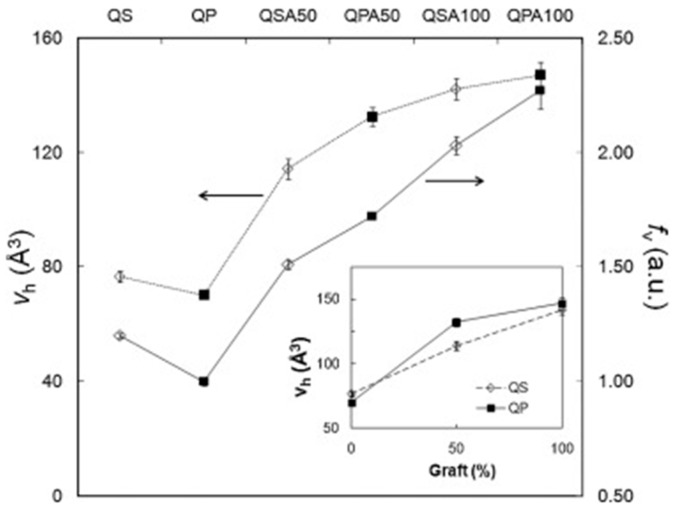
Hole free volume and fractional free volume as a function of grafting percentage for pure and grafted chitosan samples. The inset highlights the relationship between the free hole volume and grafting percentage. A more detailed description of the samples is available in ref. [81] (reproduced from ref. [81] with permission. Copyright {2016} from Elsevier).

Anbinder et al. [81] report results on the study of the synthesis and characterization of a natural/synthetic hybrid material, specifically chitosan-graft-poly(n-butyl acrylate) copolymer (pBuA). Two chitosan polymers, differing in degrees of deacetylation (DD%) and molecular weights, were grafted with pBuA using radical initiation in a surfactant-free emulsion system. Acrylate was selected for its low *T_g_* and hydrophobic properties, which could enhance chitosan’s water resistance and mechanical properties. Samples with CS:pBuA ratios of 1:0.5 and 1:1 were studied using FT-IR, DSC, Transmission Electron Microscopy (TEM), Dynamic Light Scattering (DLS), water swelling, contact angle measurements, and PALS. FT-IR confirmed successful pBuA grafting onto chitosan, with high efficiency influenced by chitosan’s DD%. TEM images (see Figure 9) showed a core–shell morphology for QSA50 grafted sample (CS:pBuA weigth ratio 1:0.5). Water swelling tests indicated enhanced water resistance, while PALS revealed an increase in hole free volume with higher grafting ratios (see Figure 10). This behavior was attributed to broken H-bonds between chitosan polar groups (NH_2_, OH) due to co-monomer grafting. The authors concluded that successful pBuA grafting improved the water resistance and mechanical properties of chitosan, with the interaction between chitosan and the grafted acrylic component contributing to enhanced performance of the chitosan-based samples.

**Figure 11:**
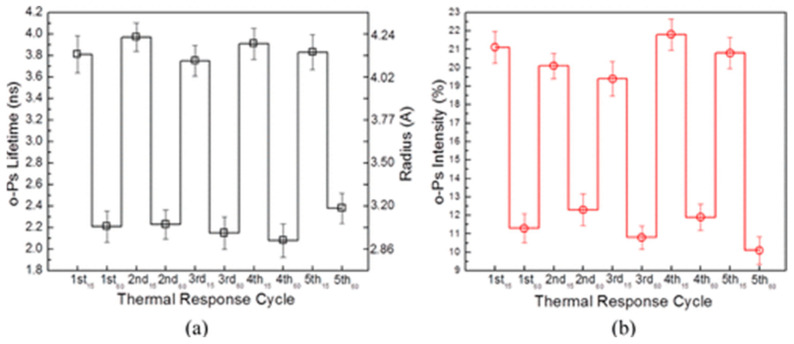
Influence of thermal cycling temperature between 15 °C and 60 °C on PALS parameters: (**a**) o-Ps lifetime (left *y*-axis) and free volume size (right *y*-axis) and (**b**) *I_o-Ps_* for the sample CS-6 mL BGE-3. A more detailed description of the samples is available in ref. [80] (Reprinted with permission from {R.L.G. Lecaros, Z-C. Syu, Y-H Chiao, Sand R. Wickramasinghe, Y-L. Ji, Q.-F An, W-S. Hung, C-C Hu, K-R. Lee, J-Y. Lai Environ. *Sci. Technol.* **2016**, *50*, 11935−11942}. Copyright {2016} American Chemical Society).

Lecaros et al. [80] investigated the synthesis of a thermoresponsive chitosan derivative created by reacting chitosan with butyl glycidyl ether (BGE) (BGE content between 1 mL and 9 mL) in an aqueous solution. This work aimed to develop a novel draw solute for forward osmosis applications, which could enhance water desalination processes while maintaining biocompatibility and nontoxicity. The successful incorporation of BGE into the chitosan structure was confirmed using FT-IR and X-ray Photoelectron Spectroscopy (XPS). UV-Vis spectroscopy confirmed the thermoresponsive behavior of the derivative, with a determined lower critical solution temperature (LCST). The LCST of the resulting chitosan derivative was determined through turbidity measurements using UV-Vis spectroscopy. The results revealed a phase transition behavior in the derivative under temperature changes. SEM and Dynamic Light Scattering (DLS) techniques were employed to analyze the particle size and morphology. The experimental results showed small and well-dispersed particles at temperatures lower than LCST, while particle aggregations were observed at higher temperatures. PALS was used to assess the free volume characteristics of the chitosan derivative during temperature cycling between 15 °C and 60 °C, showing a greater free volume at lower temperatures compared to higher ones. Thermal cycling showed reversible free volume changes (see Figure 11). At low temperatures, hydrophobic parts were hidden within well-dispersed particles, while at high temperatures, chains rearranged, exposing hydrophobic moieties and leading to aggregation and smaller hole free volumes.

**Figure 12:**
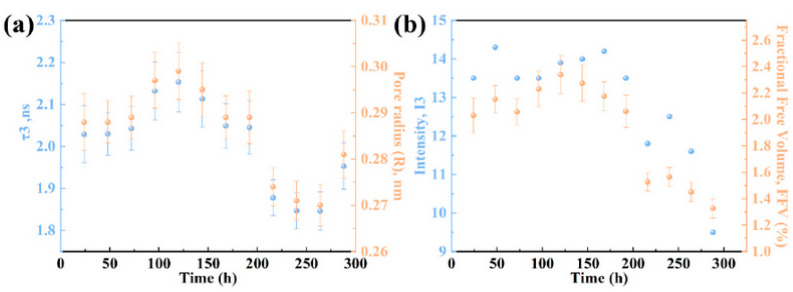
(**a**) o-Ps lifetime and pore radius; and (**b**) *I_-oPs_* and fractional free volume. All parameters were represented as a function of the release time. Values of the different parameters were obtained by measuring the sample ETH/CS-5 (0.3 mL of ethephon solution). A more detailed description of the samples is available in ref. [89] (reproduced from ref. [89] with permission. Copyright {2022} from Elsevier).

Yao et al. [89] developed a chitosan-based film (ETH/CS) for non-destructive ethylene gas sensing and controlled release. The goal of this work was to control on-demand fruit ripening, taking also into account effects related to pesticide residues associated with traditional ethephon treatments. PALS, AFM, and Raman spectroscopy were used to characterize the film’s microstructure and ethylene release mechanism. The sustained release of ethylene was monitored over time, revealing that the gas generated from the ethephon decomposition created micro-cavities within the film. PALS was used to characterize changes in hole free volume and pore size. The spectra showed variations in *τ_o-Ps_* and the associated I_o-Ps_ over time, reflecting changes in free volume size. Free volume increased initially as ethylene accumulated, peaking at 120 h before decreasing as gas was released. This behavior was associated with the formation and subsequent reduction in cavity structures in the film. The fractional free volume showed a similar trend (see Figure 12), correlating with ethylene release, which was initially high but stabilized over time. The ETH/CS film effectively controlled fruit ripening and provided a fresh-keeping effect, reducing deterioration compared to ethephon solutions.

### 3.3. Plant Oil-Based Polymers

To our knowledge, only a few papers report PALS studies on the molecular organization of polymers derived from vegetable oils [92,93,94,95].

In the literature, studies on the aging process of vegetable oil-based polymeric systems were usually carried out using different analytical techniques such as FT-IR, along with mechanical and thermomechanical analysis. However, these techniques do not provide molecular-level insights into aging processes, which PALS can offer.

**Figure 13:**
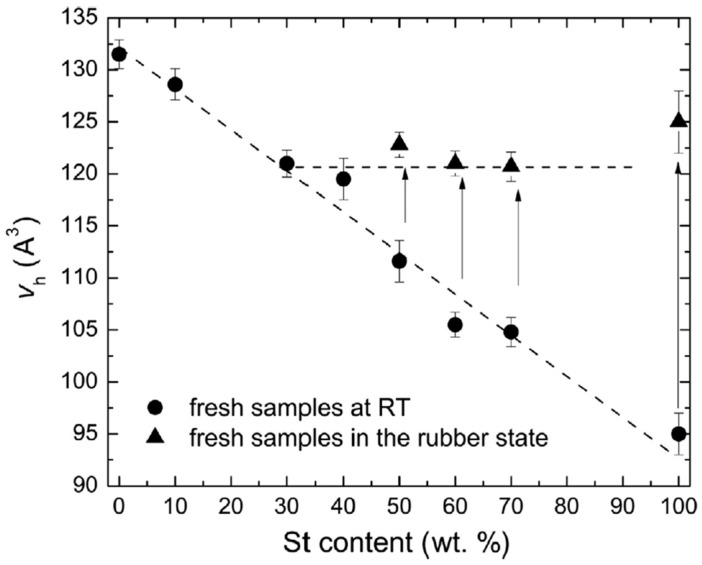
Evolution of hole free volume size with increasing St content in TO–St copolymers in the fresh state. Solid circles represent measurements at RT, while solid triangles represent measurements above T_g_. Arrows indicate changes in hole volume upon transitioning to the rubbery state (reproduced from ref. [93] with permission. Copyright {2017} from Elsevier).

Macchi et al. et al. [93] studied the effects of composition and chemical aging on tung oil–styrene networks (TO–St) synthesized through cationic polymerization. For this purpose, PALS and dynamic mechanical analysis (DMA) were used. Results on the hole free volume, *T*_g_, and dynamic mechanical properties (storage and loss moduli) were reported. It was observed that increasing St concentration within the copolymer led to a decrease in hole free volumes; this behavior was attributed to a reduction in dangling chains and an improved packing of the network structure, both reflected in a *T*_g_ increase (see Figure 13).

**Figure 14:**
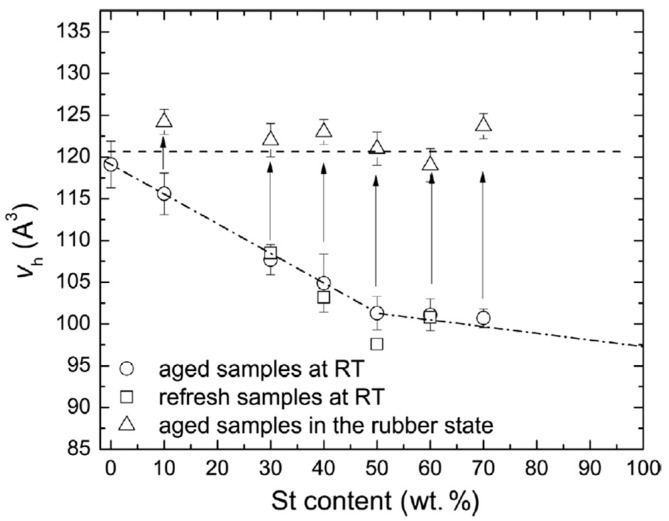
Evolution of hole free volume size with increasing St content in TO–St copolymers in the aged state. Open circles represent measurements at RT, open triangles represent measurements above *T*_g_, and open squares represent measurements of refreshed samples at RT (see more details in ref. [93]. Arrows indicate changes in hole volume upon rubbery state transition (reproduced from ref. [93] with permission. Copyright {2017} from Elsevier).

**Figure 15:**
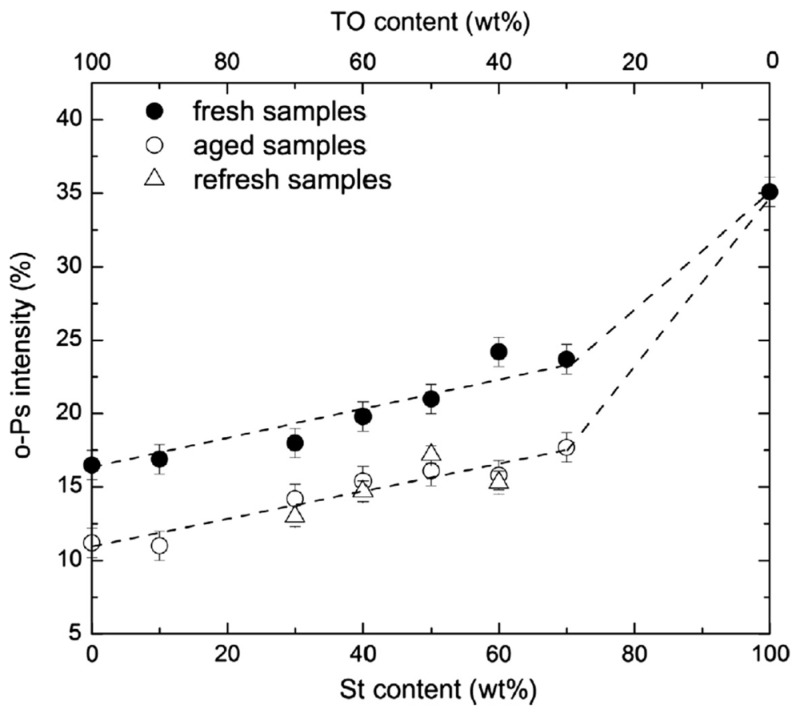
Effect of styrene content and aging on *I*_o-Ps_ in TO–St copolymers. Solid circles represent fresh samples at room temperature, open circles represent aged samples at room temperature, and open triangles represent refreshed samples at room temperature (see more details in ref. [93]) (reproduced from ref. [93] with permission. Copyright {2017} from Elsevier).

Aging decreased the free volume and increased *T*_g_ due to oxidative polymerization and degradation of the TO segments (see Figure 14). An increase in the St content led to smaller free volume but with similar values for fresh and aged samples in the rubbery state. These results indicate that the mobility of St segments allows the recovery of original hole sizes. *I_o-Ps_* values for TO-St copolymers with varying St content in the fresh, aged, and refreshed states are shown in Figure 15. The intensity increases for all sample states with increasing St content, with the highest values observed for fresh samples. Conversely, aged and refreshed samples do not show significant differences.

**Figure 16:**
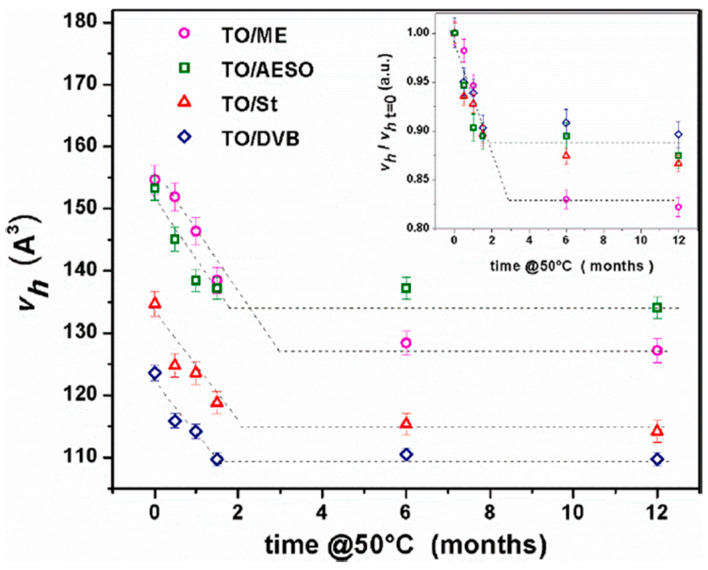
Evolution of hole free volume with aging time at 50 °C for different TO-based networks. The inset presents normalized hole free volumes (each data point normalized to its initial value at t = 0) (reproduced from ref. [95] with permission. Copyright {2020} from Elsevier).

Some of the authors studied the formulation effects and aging behavior in cationically polymerized TO thermosets containing different comonomers: the synthetic styrene (St) and divinylbenzene (DVB); the bio-based comonomer tung oil methyl ester (ME); and the bio-based modifier acrylated epoxidized soybean oil (AESO) [95]. In all cases, a fixed TO/comonomer ratio of 70/30 was used. FT-IR, DMA, PALS, and TGA were employed to study chemical aging effects on *T*_g_, storage moduli, and hole free volume. Results indicated that the comonomer type influenced these parameters. Aging increased *T*_g,_ and decreased *v*_h,_ due to oxidative polymerization and degradation processes in the polymer thermosets. PALS showed a decreasing *v*_h_ with aging time, indicating an unstable network. Mild heating at 50 °C accelerated aging, leading to significant *v*_h_ reduction within the first two months (Figure 16). Oxidative processes likely caused crosslinking and the loss of volatile compounds, resulting in densification of the polymeric matrices.

## 4. Conclusions

The growing attention to environmental issues and the increasing demand for eco-friendly products have made research about natural resource-based polymers a popular topic for researchers. These polymers are seen as potential substitutes for synthetic materials, offering a more sustainable solution. To develop high-performance natural polymers, it is essential to have a deep understanding of their properties and how they respond to various chemical, physical, and mechanical stimuli. The concept of free volume is closely linked to key properties of a polymer. Indeed, as discussed above, there is a strong correlation between the mechanical and transport properties of polymeric structures, as well as their aging processes, and the amount of free volume present.

The examples presented in this review, which focus on the study of the modifications in the molecular packing of green-based polymers through changes in synthesis or environmental conditions, highlight the value of PALS as a technique for probing free volume at the nanoscale within polymeric structures. This technique allows researchers to investigate holes located among macromolecular chains. In a broader context, extending beyond green polymers to all polymeric materials, the direct information on the free volume provided by PALS offers valuable insights into the molecular organization of polymers. This knowledge can facilitate the design and synthesis of polymers with enhanced properties.

Due to the limited length of this article, we have focused on a few specific categories of natural polymers, some of which we have previously studied. However, it is important to note that, in principle, any polymeric structure can be examined using PALS, as long as positronium can form inside it.

Future investigations will benefit from comparing free volume measurements obtained by PALS with molecular simulations. While simulations are not widely used today due to computational limitations, they hold the potential to verify PALS results and provide additional information to deepen our understanding of molecular organization within a polymer.

In conclusion, advancements in positron annihilation techniques will enhance our understanding of free volume, a crucial parameter that influences many polymer characteristics but that cannot be directly measured using conventional experimental techniques.

## Data Availability

No new data were created or analyzed in this study. Data sharing is not applicable to this article.
